# How do we evaluate outcome in an integrative oncology program?

**DOI:** 10.3747/co.v15i0.271

**Published:** 2008-08

**Authors:** S.M. Sagar

**Keywords:** Integrative, oncology, audit, measurement, outcomes, quantitative, qualitative, economics, services

## Abstract

Integrative oncology focuses on the roles of complementary therapies to increase the effectiveness of conventional cancer treatment programs by improving defined outcomes such as symptom control, quality of life, rehabilitation, and prevention of recurrence. Implementation of integrative oncology programs should be based on the best evidence and must continually be evaluated to ensure quality, optimization of techniques, collection of new data, and cost-effectiveness. Useful domains that can be evaluated include symptom control, adherence to treatment protocols, quality of life, individual outcomes, prevention, rehabilitation, potential advantages of a whole-systems health approach, and economics of health services.

## 1. INTRODUCTION

Integrative oncology focuses on the roles of complementary therapies such as meditation and other mind–body approaches, music therapy, massage and other touch therapies, acupuncture, natural health products (botanicals, for example), nutrition, fitness therapies, and more. Its goal is to increase the effectiveness of conventional cancer treatment programs, to reduce symptoms, and to improve quality of life for cancer patients. The Consortium of Academic Health Centers for Integrative Medicine defines integrative medicine as “the practice of medicine that reaffirms the importance of the relationship between practitioner and patient, focuses on the whole person, is informed by evidence, and makes use of all appropriate therapeutic approaches, healthcare professionals and disciplines to achieve optimal health and healing.”

## 2. IMPLEMENTING INTEGRATIVE ONCOLOGY

Implementation of integrative oncology programs should be based on the best evidence and must continually be evaluated to ensure quality, optimization of techniques, collection of new data, and cost-effectiveness. Useful domains that can be evaluated include symptom control, adherence to treatment protocols, quality of life, individual outcomes, prevention, rehabilitation, potential advantages of a whole-systems health approach, and economics of health services.

### 2.1 Symptom Control

Various modalities play a role in symptom control. These include mind–body therapies (meditation, group support, cognitive behavioural therapy, spirituality, yoga, tai chi, *qi gong,* labyrinth walking), manipulative and physical therapies (massage, acupuncture), biofield therapies (polarity, healing touch, Reiki), natural health products (botanicals), nutrition interventions (phytoceuticals, diet adjustment), and ethnic health systems (Traditional Chinese Medicine, Ayurveda, Native American Medicine) 1. Current evidence for safety and effectiveness has been summarized by the Society for Integrative Oncology 2. Visual analogue scales are often used to measure such symptoms as pain, anxiety, and tension at baseline and after treatment. Validated assessment procedures are required to select and triage specific patients to an appropriate therapy program.

An integrative medicine program provides opportunities to connect with the patient, enhance trust, and communicate information in a supportive environment. These factors increase adherence to treatment protocols and can improve treatment response and survival 3,4. Positive expectation, appreciation of meaning, and a sense of optimism should be enhanced within the context of factual knowledge 5,6.

A National Health System report from the United Kingdom encourages positive expectation in the delivery of health care. A pessimistic approach can encourage the nocebo effect, which can result in poor coping skills, disempowerment, and adverse health outcomes 7. The aim of the report was to assess the nature and extent of the placebo effect and to consider how it may be harnessed within the National Health System to improve the quality of care. The authors concluded that the existing evidence justifies the use of strategies to enhance expectancies—specifically, to enhance accurate expectations by the patients about medical procedures and how to cope with them. In addition, evidence encourages the use of the positive effects of expectation to enhance the skills of patients for self-management of their illness, to improve communication concerning health issues with health care providers, and to enhance belief in the benefits of effective medical treatments.

### 2.2 Patient-Focused Approach

Patients that choose complementary or alternative therapies require guidance. Such guidance is often not available in cancer treatment organizations because many of the staff are not trained in integrative oncology and because information resources have not been critically examined by a qualified person for credibility. Cancer “guide,” “navigator,” or “pathfinder” is an important role for an appropriately trained health care professional. Although many patients are simply seeking empowerment and trying to do everything possible, some patients have important psychosocial issues that need to be diagnosed and managed appropriately. These issues can include cultural beliefs, heightened anxiety, fear, anger, advanced and uncontrolled symptom burden, poor satisfaction with conventional medicine, communication breakdown, and misinformation that includes bogus therapies found on the Internet 8,9.

The evaluation of integrative health care models is becoming increasingly important, and an appropriate set of outcome measures is required. Dr. Marja Verhoef has proposed these questions 10:

- How do we identify the manner in which cancer patients phrase and frame the beneficial outcomes of their experience?
- What are the recommendations that can be made for an appropriate outcome measures package to evaluate integrative health care?

Through interviews and focus groups, Verhoef identified six types of benefit: physical well-being, change in physiologic indicators, improved emotional well-being, personal transformation, feelings of connectedness, global state of well-being, and cure. Patients’ goals included improvement in their state of being, freedom from cancer, increased energy, more effective pain management, and achievement of an improved quality of life.

The evaluation of medical practice should use quantitative and qualitative techniques alike. Contemporary medicine is assumed to be conceptually based on modern science; however, the summation of quantity and quality is impossible to evaluate using only scientific parameters. Integrative medicine is postulated to be comprehensive in its fundamental doctrine, emphasizing a holistic approach, including technical, artistic, social, religious, philosophic, and ethical elements. The personal preferences of patients for outcome as defined by qualitative experiences is emphasized. The World Health Organization suggests that health be evaluated from the viewpoint of the disability-adjusted life expectancy, which is expressed using both quantity (life expectancy) and quality of life. One of the challenges of assessing the utility of integrative oncology is the expression of joint measurements of quantitative and qualitative outcomes. Medical quantity and quality are in reality mixed and fused together, both in treatment and in outcomes.

#### 2.2.1 Quantitative Evaluation of an Intervention for an Individual

The N-of-1 randomized controlled trial was designed to evaluate the contribution to the therapeutic process of an intervention as compared with either a placebo (expectation) effect or regression to the mean. The patient participates in choosing the intervention. Distinguishing the components of healing is probably not an important issue if the intervention is safe and inexpensive. It may not be applicable to the cancer patient when conditions change rapidly. In addition, the resources and expenses of implementing this type of evaluation must be taken into account. That being said, the N-of-1 trial may have a place in the evaluation of some treatment programs 11,12.

#### 2.2.2 Qualitative Evaluation of an Intervention for an Individual

A major benefit of integrative therapies is their potential to improve quality of life (qol). Evaluation of qol is now a standard of clinical investigation that is routinely used in addition to measurement of tumour response. An improvement in symptom control, coping, and function enhances rehabilitation and restoration of a normal lifestyle. Nevertheless, an understanding of what qol is and how it should be evaluated is a very difficult matter.

A fundamental part of the definition of a high qol is a large degree of freedom in thinking and behaviour that includes personal subjective feelings. As a result, the cornerstones of science—which include objectivity, universality, reproducibility, and logical consistency—can no longer be totally applied. Evaluation of medical practices in terms of qol is non-scientific in this respect, because those scientific characteristics are not preserved 13. The fact that so many qol evaluation parameters have been proposed ultimately suggests that none are reliable. Unless a logical and scientific way of assessing personal feelings is established, qol simply cannot be evaluated using scientific analysis and numeric expression.

Because qol may vary between individuals according to their primary problems and values, individual and personalized qol criteria may be more appropriate for measuring outcomes. The Measure Yourself Medical Outcome Profile was developed to allow patients to set their own criteria for qol outcomes. This measure was developed further for cancer patients in the form of the validated Measure Yourself Concerns and Wellbeing assessment protocol, which specifically evaluates outcome in cancer supportive care that includes complementary therapies 14,15 (Table I).

The term ‘‘healing enhancement’’ (Mayo Clinic, Rochester, MN, U.S.A.) was coined to identify the goals of an emerging paradigm that focuses on all aspects of the patient’s experience—mind, body, and spirit 16. That subjective experience of healing was investigated in a multi-method pilot study 17: Before and after each of 6 weekly healing sessions, perception of well-being and client experience were assessed in 15 clients by the EuroQol, the Measure Yourself Concerns and Wellbeing index, and a client satisfaction tool. Qualitative methods included focus groups that explored the perceived effects of healing in more depth. Over the course of healing, quantitative data showed perceived significant improvements in concerns or problems for which clients wanted help and in distressing symptoms of anxiety and depression. Qualitative analysis showed that clients sought help mainly for psychological and emotional concerns. Clients attributed many of the quantitative improvements to healing itself. The study suggested that clients and healers perceive healing to have a range of benefits, particularly in terms of coping with cancer.

Research to date indicates that patients who are involved in their own care and who take an active role in their treatment feel better and have an improved recovery. Other studies have shown that depression predicts cancer incidence or progression, although not all studies agree. Rosenbaum *et al.* 18 discussed this effect in more detail. A randomized study that compared qol according to the survival of patients in the adjuvant and metastatic settings noted that an increase in qol included improved mood, appetite, and well-being, and hence facilitated longer survival 19. That finding suggests that qol may also play a role in determining a patient’s length of life. Resources and programs that are shown to improve qol, such as the Stanford Cancer Supportive Care Program, promote a more meaningful and longer lifespan for those struggling with cancer.

Because qol involves subjective measurement in domains such as the psychological, functional, and social, it can be difficult to assess. Unlike readily quantifiable measures, such as weight or tumour size, qol is not as easily defined. Interpretation of qol differs with each person and is influenced by factors such as culture, age, and life experience. The patient evaluations from the Stanford Cancer Supportive Care Program suggest that participants received several benefits from the programs, such as an increase in sense of well-being, reduced feelings of stress, an increase in energy, more restful sleep, and an increase in hopefulness and empowerment—all ingredients that improve qol 18.

### 2.3 Systems Approach to Health Care

Integrative oncology recognizes a systems approach to health care. A program of coordinated appropriate interventions may be more effective than individual therapies. The holistic approach to health is a maturing tautology that is now binding the sciences from molecular biology to psychosocial and health services research 20. Systems approaches, by taking the myriad of connections into account, are more suited for addressing chronic, long-term improvements. In many chronic diseases, the body system may not have been healthy over a prolonged period of time before the symptoms appeared.

Only short-term improvements can be obtained by aiming at a particular subsystem related to the symptoms. Overall sustained improvement can be obtained only through multiple interventions. A multi-pronged approach encourages homeostasis to return to the whole physiologic organism. For example, diabetes care involves the interaction of nutrition, exercise, stress management, and education—not just the prescription of insulin. Instead of disease management, health promotion becomes the aim of the intervention.

Whole-systems research explores effectiveness through observational studies that combine qualitative and quantitative approaches such as pattern of interactions, context, process, philosophy, and outcomes. It deals with “real life” scenarios and recognizes multiple inseparable components, model validity, effectiveness, and external validity 21,22. A systems analysis can reveal new approaches to improve the current situation of ever-increasing health care costs.

### 2.4 Health Economics

The financial implications of integrative oncology programs require evaluation with respect to functional outcomes, in-kind contributions, safety, restoration of function, and efficiency of rehabilitation 23. The self-empowerment of individuals using complementary therapies can have life-long advantages for self–health care. Short-term measurements of outcome may not reflect lifetime benefits 24. Using complementary therapies over the long term, together with judicious temporary pharmacologic therapies, could have cost-effectiveness advantages. For example, short-term administration of antidepressant drugs may give the patient adequate function to learn cognitive behavioural techniques that will become the source of a maintenance regimen after withdrawal of the pharmaceuticals.

Measuring qol with respect to financial cost is methodologically challenging. Quality-adjusted life year (qaly) is a quantitative measurement that may allow for a financial comparison between various interventions and their related health outcomes. The main problem is to be able to compare “apples with oranges” (to use a common expression). The ability to compare directly the dollar cost of various health outcomes is attractive to the decision-maker. However, the use of the qaly for this purpose has severe limitations, and these limitations must be widely understood 25. A key question is who is to make the subjective choices that determine the qaly? Is it health professionals? The general public? Or patients who have experience of the particular medical condition and treatment? Other problems include the hypothetical nature of the situations being responded to, which therefore may not accurately reflect real human decisions, and the influence that the length of the illness and the way in which the questions are asked influence the valuations made. Finally, qalys are likely to undervalue health care because they do not capture the wider benefits.

The evidence for cost advantages of complementary therapies is limited because the research is at an early stage 26,27. Many outcomes studies are not well controlled, and determining whether outcome is attributable to the intervention, expectation, regression to the mean, or a combination of these factors is difficult. This difficulty has led to much controversy and many unwarranted claims 28,29. However, uncertain evidence of effectiveness does not necessarily preclude a positive recommendation in a guideline, and original modelling of cost-effectiveness can be part of guideline development 30.

## 3. CHALLENGES

Integrating complementary therapies into conventional cancer care poses a number of challenges. What is required is an integration of the best approaches to cancer care with a more cohesive move forward based on collaboration and open-mindedness. Skepticism comes from lack of knowledge and training, and the result is a reluctance to refer patients to cam therapists 31. However, implementation of an evidence-based integrative medicine program within an academic setting can be successful. A survey of one large academic institution with a Chair for Holistic and Integrative Medicine (Wake Forest University Baptist Medical Center, Winston–Salem, NC, U.S.A.) concluded that strong interest is present among medical staff. Future studies will need to assess the cost-effectiveness of such services, their financial sustainability, and their effect on patient satisfaction, health, and quality of life 32.

Most mainstream and complementary medicine practitioners agree that patients should take an active role in their care, that an understanding of treatment and its goals should be established between the patient and practitioners, and that the culture should encourage patients to take responsibility for their health 33. We need to focus on the “effectiveness gaps” in the treatment of cancer patients. For example, emesis, fatigue, neuropathy, anxiety, insomnia, and cachexia are some symptoms that continue to show problematic responses to conventional therapies. Drawing together services and developing expertise in integrative health care requires leadership to promote team-building. However, without collaboration and support, such visions are limited. Without audit and evaluation of outcomes, they will not be accepted by mainstream practitioners. Championing integrative oncology focuses on the value of the vision and the public acknowledgment of that value. A willingness to measure and act upon the results of clinical outcomes studies is more important than are policies based on outdated cynicism (Table II) 34.

**TABLE I:** Measure Yourself Concerns and Wellbeing assessment form a

Concern or problem 1:	0	1	2	3	4	5	6
	Not bothering me at all				Bothers me greatly
How would you rate your general feeling of well being now?	0	1	2	3	4	5	6
	As good as it can be				As bad as it can be
Other things affecting your health; changes you have made yourself
What has been most important to you?

^a^ Available at www.bristol.ac.uk/hsrc/research/other/mymop/index.html (registration required)

**TABLE II:** Champion ideas for cam and cancer care (adapted from Mackereth and Stringer, 2005 34)

Encourage researchers to be sensitive to the vulnerabilities of people affected by cancer and mindful of their values and concerns.
Collaborate with cancer interest groups, education and communication specialists, researchers, other national and international programs.
Provide leadership training and support to assist practitioner innovation, to build dynamic services, and to disseminate their work.
Improve information and access to cam therapies within public cancer services.
Provide greater equity in funding service provision and research.
Involve users, with the goal of providing cost-effective, accountable, and responsive services.
Ensure safety of therapies and credentialing of practitioners.
